# Nebulized epinephrine for croup in children: an updated systematic review

**DOI:** 10.3389/fped.2026.1886410

**Published:** 2026-08-26

**Authors:** Yonggang Wei, Lei Li, Nian Wei, Yuanlin Pu, Jihua Zhao, Ping Xiong

**Affiliations:** Department of Pediatrics, The Central Hospital of Enshi Tujia and Miao Autonomous Prefecture, Enshi, China

**Keywords:** children, croup, epinephrine, meta—analysis, systematic review

## Abstract

**Objective:**

To review and update the clinical evidence on the use of nebulized epinephrine for croup in children.

**Methods:**

The protocol was registered with PROSPERO (CRD420251160013). Our search encompassed PubMed (MEDLINE), Embase, Web of Science, Scopus, the Cochrane Library databases for randomized controlled trials (RCTs) investigating epinephrine for the treatment of croup. The search covered the period from database inception to July 3, 2026. We also searched two clinical trials registries: the World Health Organization International Clinical Trials Registry Platform (WHO ICTRP) and clinicaltrials.gov (searched 03 July 2026). We included studies that reported epinephrine in the treatment of croup in children. This systematic review was reported in accordance with the PRISMA 2020 guidelines.

**Results:**

A total of 12 studies were included in the analysis, with 325 patients in the treatment group and 347 in the control group. The meta-analysis revealed that nebulised epinephrine significantly improved croup severity score at 30 min post-administration (SMD = −1.48, 95% CI [−2.83, −0.13]). However, this effect was not sustained. At 2 h, the between group difference was no longer statistically significant (SMD = −0.51, 95% CI [−1.11, 0.09]). Regarding hospital stay, although the point estimate suggested a clinically meaningful reduction (MD = −25.1 h, 95% CI [−52.34, 2.15]), the wide confidence interval crossed zero and the difference did not reach statistical significance (*P* = 0.07).

**Conclusion:**

Nebulized epinephrine significantly improved croup score at 30 min, but its sustained effect at 2 h remains inconclusive due to unstable sensitivity analysis. No significant reduction in hospital stay was observed (*P* = 0.07). Routine use for shortening hospital stay is not supported by current evidence, although it remains a valuable early intervention in moderate-to-severe acute settings.

**Systematic Review Registration:**

https://www.crd.york.ac.uk/PROSPERO/recorddashboard, PROSPERO CRD420251160013.

## Introduction

Croup is a common childhood disease characterized by the sudden onset of a distinctive barky cough, usually accompanied by stridor, hoarse voice, and respiratory distress due to upper airway obstruction (1). The condition predominantly affects children between 6 months and 3 years of age, with a peak incidence during autumn and winter nights (2). Most cases are caused by viral infections (3). Parainfluenza virus is the most commonly identified (4). The hospitalization rate for croup is less than 5% −7% (1, 3). However, among admitted patients, 1% to 3% require intubation (1, 3). Therefore, despite its low hospitalization rate, croup remains a clinically significant condition that warrants careful management.

Glucocorticoids are currently widely used in the management of croup (5, 6). However, their onset of action is relatively slow (7). Although epinephrine has long been recommended for the rapid relief of upper airway obstruction (8, 9), its clinical application has been constrained by two practical issues. First, racemic epinephrine is not readily available in many regions. Second, the use of L-epinephrine via nebulization constitutes off-label use, as this route of administration is not included in the package insert.

This study aims to update the evidence on the efficacy and time course of epinephrine in children with moderate-to-severe croup. Based on the observed temporal patterns of clinical response, we provide a systematic evaluation of its role as an early acute intervention, and critically assess whether this transient improvement translates into a reduced hospital length of stay.

## Method

### Study registration and reporting guidelines

The primary objective of this study is to evaluate the efficacy and adverse reactions of epinephrine in the treatment of croup in children. This study was registered in the PROSPERO International Prospective Register of Systematic Reviews (Registration number: CRD420251160013). This study was conducted following the PRISMA 2020 guidelines (10).

### Search strategy

We searched PubMed, Embase, Cochrane Library, Scopus, and Web of Science from inception to July 3, 2026, with language restricted to English. The search strategy employed a combination of Medical Subject Headings (MeSH) and free-text terms (e.g., “croup OR laryngitis” AND “epinephrine OR adrenaline”). Manual searches of the references from relevant articles were also conducted to ensure comprehensiveness. A detailed account of the search strategy is provided in Supplementary Table 1. In addition, we searched two clinical trials registries: the World Health Organization International Clinical Trials Registry Platform (WHO ICTRP) and clinicaltrials.gov (searched 03 July 2026).

### Definitions of key terms

Croup score refers to a class of clinical tools used to quantify the severity of airway obstruction and clinical symptoms in patients with acute croup. These scores typically grade or assign points based on signs such as stridor, respiratory difficulty, chest retractions, facial color, and level of consciousness. Common specific tools include the Westley Croup Score (11), the Modified Taussig Croup Score (12), the Downes and Raphaely Croup Score (13), and the pediatric acute laryngitis dyspnea grading system (14). A higher score or grade indicates more severe airway obstruction.

### Inclusion and exclusion criteria

Inclusion criteria:

Population: Pediatric patients diagnosed with acute croup.

Design: RCT.

Intervention: Comparison of nebulized epinephrine (racemic or L-epinephrine) vs. control (e.g., nebulized normal saline/budesonide or intramuscular dexamethasone or others).

Outcomes: At least one quantitatively reported prespecified outcome, including but not limited to severity score at 30–120 min, and length of hospital stay.

Exclusion criteria:

Involve patients with non-viral etiologies of upper airway obstruction, such as laryngeal foreign body aspiration or post-intubation laryngitis.

Are case reports, editorials, commentaries, or review articles.

Utilize non-randomized study designs (e.g., cohort studies, case-control studies).

Are published in languages other than English.

### Study selection and data extraction

Two researchers (YW and LL) conducted independent literature searches and data extraction. During the screening process, the established inclusion and exclusion criteria were rigorously adhered to. In instances where there was a divergence of opinion, the input of experts was sought (PX and JZ). The relevant indicators from the studies were extracted and cross-checked to ensure consistency. The primary data extraction content included the first author, year of publication, country, study population, sample size, drug name, dosage, and administration method.

### Quality assessment

Two reviewers (YW and LL) independently assessed the risk of bias for each included randomized controlled trial using the Cochrane Risk of Bias Tool. The tool evaluates seven domains: random sequence generation, allocation concealment, blinding of participants and personnel, blinding of outcome assessment, incomplete outcome data, selective reporting, and other potential biases. Each domain was judged to be at “low risk”, “high risk”, or “unclear risk”. Any disagreements were resolved by consensus or by consultation with a third reviewer (YP).

### Statistical analysis

The statistical analysis was conducted using Review Manager 5.3.5 and Stata 15. The standardized mean difference (SMD) or mean difference (MD) with 95% CI was used depending on whether the same measurement scales or units were used across studies. Pooled estimates were derived from a fixed or random effects model based on heterogeneity. *P* < 0.05 was considered significant. For outcomes with ≥10 studies, publication bias was assessed using funnel plots and Egger's test. To assess the stability of the overall findings and identify the origins of bias, we conducted sensitivity analyses using Stata 15. Specifically, a “leave-one-out” approach was applied to determine whether any single study disproportionately influenced the pooled effect estimates. For studies that did not provide extractable numerical data (e.g., reported only as categorical response rates, medians with ranges, or narrative descriptions without means and standard deviations), a narrative synthesis was performed instead of quantitative pooling.

## Results

### Literature search results

**Figure 1:**
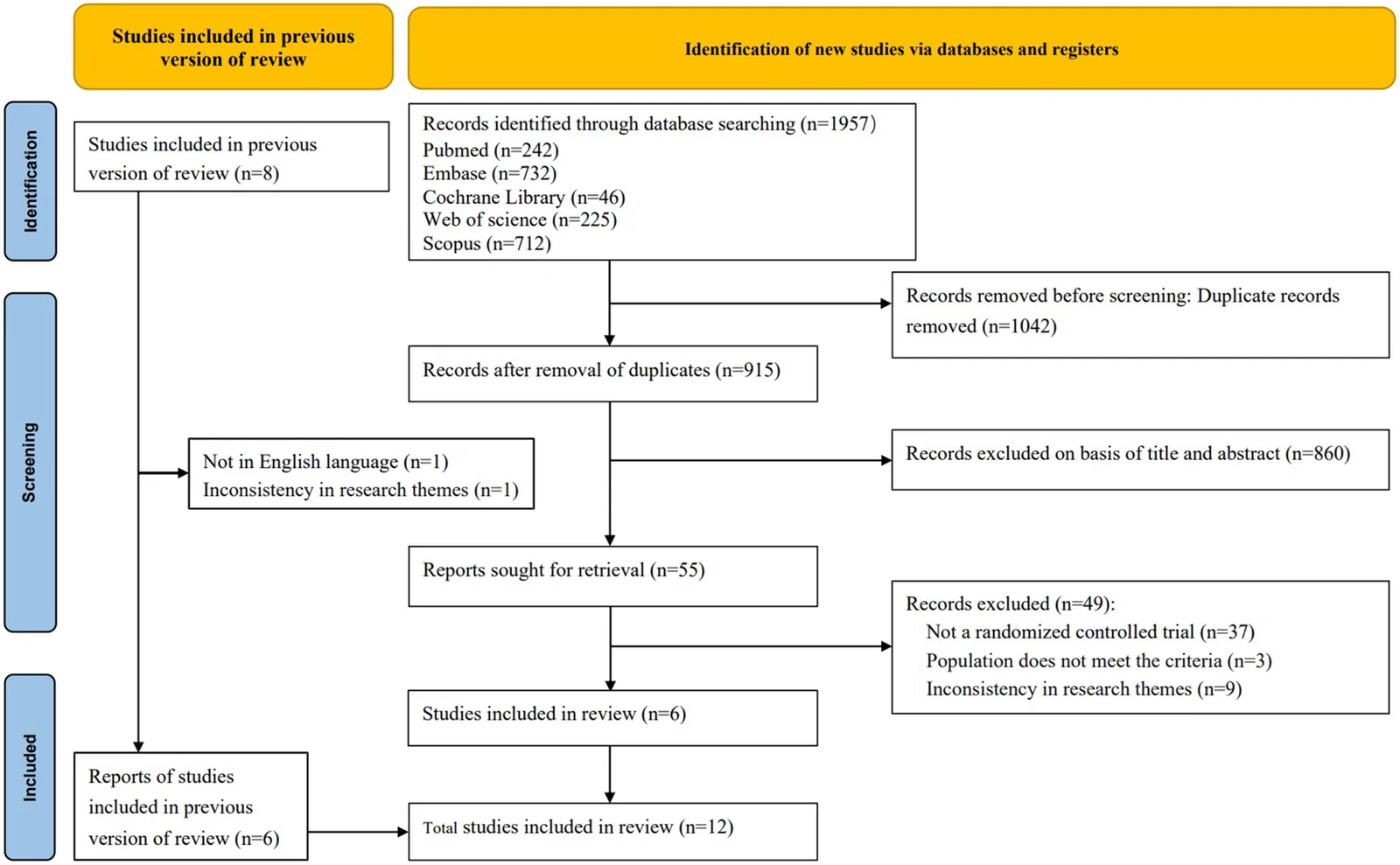
Preferred reporting items for systematic reviews and meta-analyses (PRISMA) flow diagram.

A total of 1,957 articles were identified from initial searches in PubMed, Embase, the Cochrane Library, Scopus, and Web of Science. After deduplication, 915 articles remained. Following a review of titles and abstracts, 55 articles were selected for full-text screening, from which 6 studies (11, 12, 15–18) met the inclusion criteria. Additionally, 6 studies (13, 14, 19–22) included in the previous version of the review (23) were retained, resulting in a total of 12 included studies (11–22). The specific search process is illustrated in Figure 1.

Registry search results: A search of the WHO ICTRP identified one published study comparing different doses of epinephrine for croup (24). In addition, a search of ClinicalTrials.gov identified three other registered trials (IRCT20241229064209N1, IRCT201105156491N1, IRCT201105264396N2), all of which were ongoing or unpublished at the time of the search.

### Characteristics of the included studies

A total of 12 studies (11–22) were included in the review, with the majority originating from USA (four studies), Canada (two studies), and one study each from the Iran, Turkey, Sweden, Australia, Finland, and China. The treatment group comprised 325 patients, while the control group consisted of 347 individuals. The characteristics of the included studies are detailed in Table 1.

**Table 1:** Baseline characteristics, disease severity, and intervention protocols of the included studies.

Author	Year	Country	Age Range	Assessment Scale	Disease Severity	Treatment Group (n)	Control Group (n)	Treatment Group Intervention	Treatment Dose	Control Group Intervention	Control Dose
Studies included in the meta-analysis											
Huazhen Wang (18)	2022	China	1y—6y	Clinical Scoring (18)	Unknow	50	50	LE and BUD	LE 1 ml, BUD 2 ml Neb	BUD	BUD 2 ml Neb
Eghbali, A (11)	2016	Iran	6 m—6y	Westley croup scores (11)	Mild, Moderate, Severe	87	87	LE and DXM	LE 0.5 mg/kg Neb, DXM 0.6 mg/kg IM (max 8 mg)	DXM and Placebo	DXM 0.6 mg/kg IM (max 8 mg)
Kristjánsson, S (20)	1994	Sweden	4 m —11y	Westley/Taussig croup scores (20)	Mild, Moderate, Severe	25	29	RE	RE 0.5 mg/kg Neb	Saline (placebo)	Saline 0.5 mg/kg Neb
Kuusela, A. L (21)	1988	Finland	6 m —11y	Symptom scoring (21)	Unknow	19	16	RE and DXM	RE.05 ml/kg Neb, DXM 0.6 mg/kg IM	DXM and Placebo	DXM 0.6 mg/kg IM
						16	21	RE and Placebo	RE 0.05 ml/kg Neb	Placebo	
Westley, C. R (22)	1978	USA	4 m—5y	Clinical Scoring (22)	Unknow	10	10	RE	RE 0.5 ml, preservative-free sterile water 3.5 ml Neb	Saline	Saline 0.5 ml, preservative-free sterile water 3.5 ml Neb
Taussig, L. M (17)	1975	Canada	5 m —11y	Clinical Scoring (17)	Moderate, Severe	7	6	RE	RE 0.5 mg/kg Neb	Supportive care	Mist ten, oxygen, hydration and antibiotics as needed.
Studies not included in the meta-analysis											
Duman, M (15)	2005	Turkey	≥6m	Westley scoring system (15)	Moderate, Severe	31	26	LE and DXM	LE 0–20 kg 2.5 mL/20–40 kg 5.0 mL Neb, DXM 0.6 mg/kg IM	Cool mist therapy and DXM	DXM 0.6 mg/kg IM
Weber, J. E (12)	2001	USA	6 m—3y	Modified Taussig Croup Score (12)	Moderate, Severe	15	14	RE and DXM	RE 0.5 ml Neb, DXM 0.6 mg/kg IM	Heliox and DXM	DXM 0.6 mg/kg IM
Fitzgerald, D (16)	1996	Australia	6 m—6y	Croup Symptom Score (16)	Moderate, Severe	31	35	LE	LE 4 mg Neb	BUD	BUD 2 mg Neb
Waisman, Y (13)	1992	USA	6 m—6y	Downes and Raphaely croup score (13)	Moderate, Severe	14	14	RE	RE 0.5 mL Neb	LE	LE 5 mL Neb
Corkey, C. W (14)	1981	Canada	≤3y	Clinical Scoring (14)	Moderate, Severe	10	10	RE	RE 0.5 ml, distilled water 3.5 ml Neb	Distilled water	Distilled water 4 ml Neb
Gardner, H. G (19)	1973	USA	5 m—5y	Clinical Scoring (19)	Moderate, Severe	10	10	RE	RE 0.5 ml, saline: 3.5 ml Neb	Placebo	Saline 4 ml Neb

RE, Racemic epinephrine; LE, L-epinephrine; DXM, Dexamethasone; BUD, Budesonide; Neb, Nebulization; IM, Intramuscular.

### Methodological quality and risk of bias

**Figure 2:**
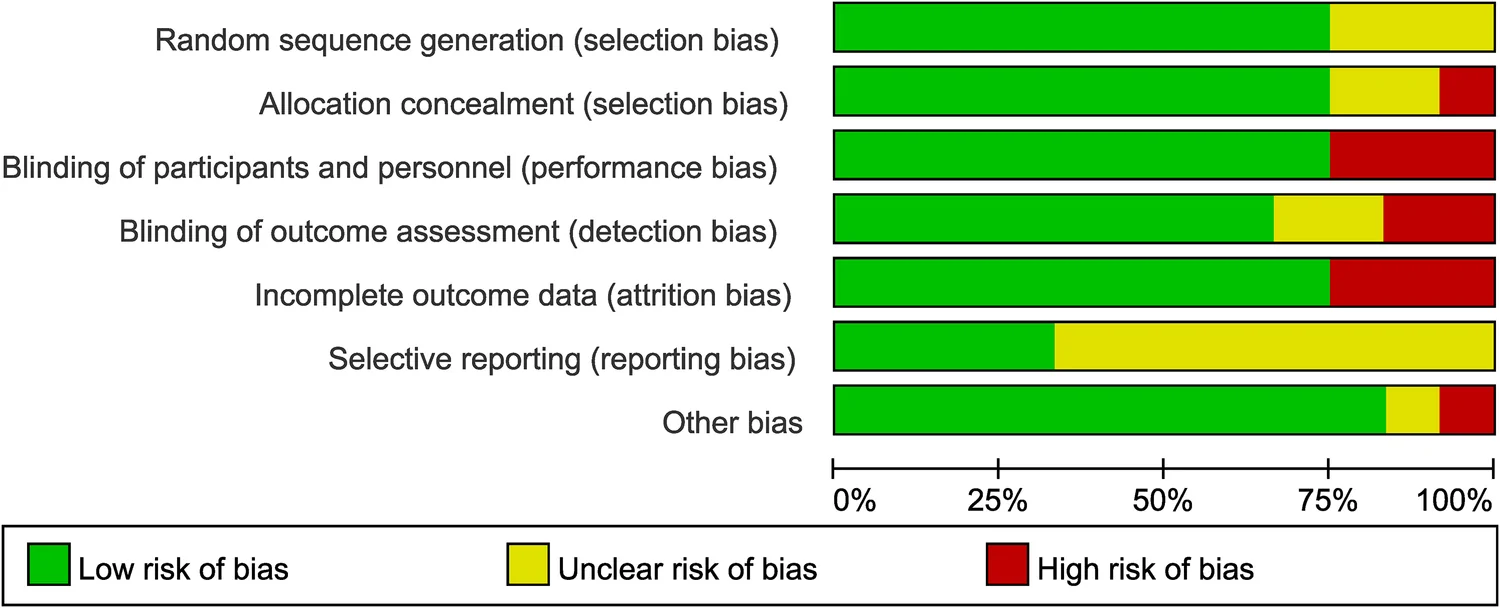
Risk of bias graph (percentages across all included studies).

**Figure 3:**
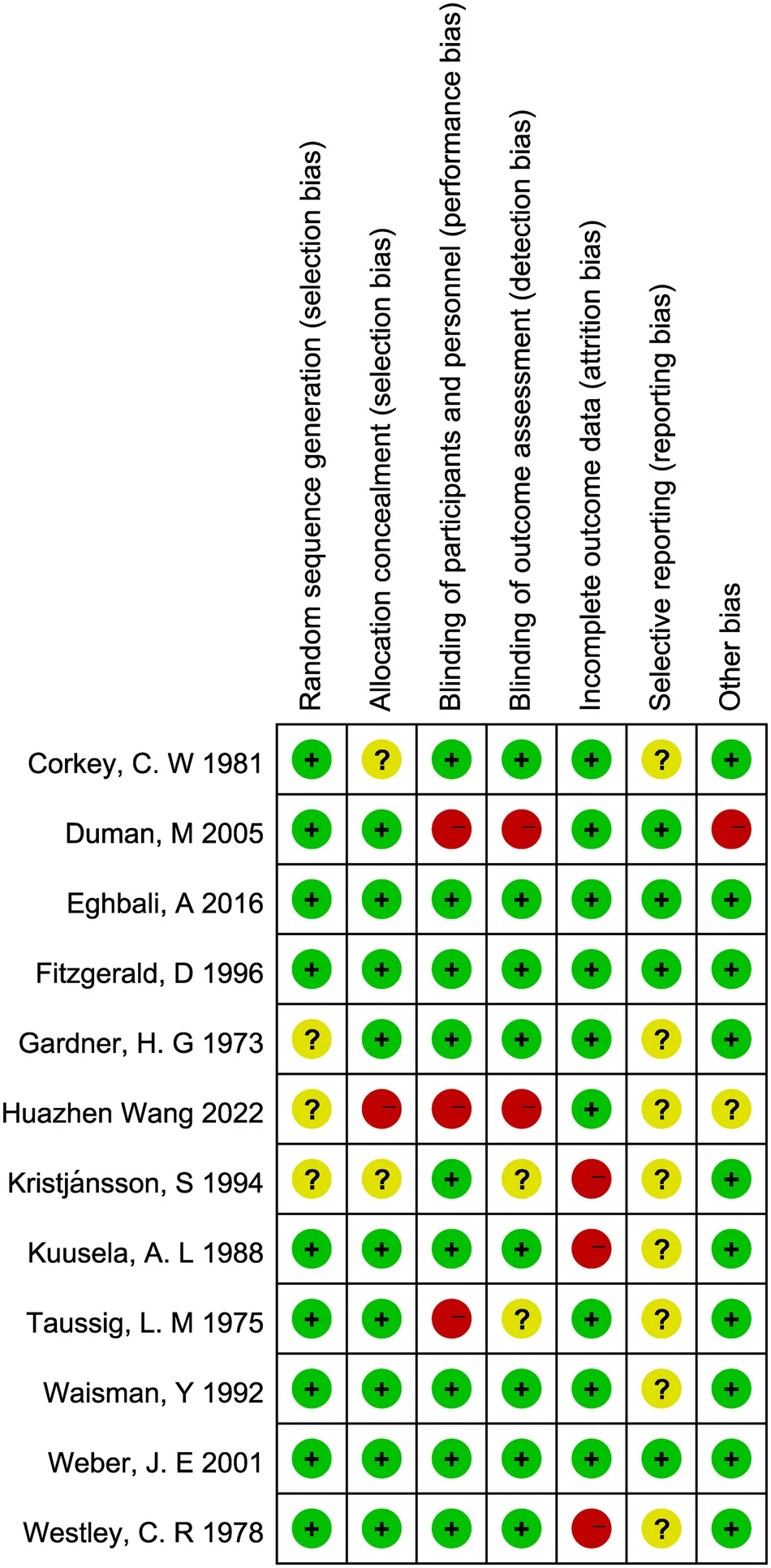
Risk of bias summary by study and item.

The Cochrane Risk of Bias Tool was utilized to evaluate the quality of the included studies. In the quality assessment, three studies required adjustments to treatment regimens due to changes in the subjects' conditions, leading to missing data. Furthermore, a high proportion of studies were rated as having a high risk of bias for blinding, primarily because the methods of drug administration were inconsistent across some studies. The specific quality ratings are presented in Figures 2, 3.

### Outcomes

#### Croup score

To account for the disparate scoring systems across studies, we used the SMD for data synthesis. We assessed croup scores at two post-intervention time points: 30 min (11, 17, 20, 22) and 120 min (11, 17, 21, 22).

**Figure 4:**
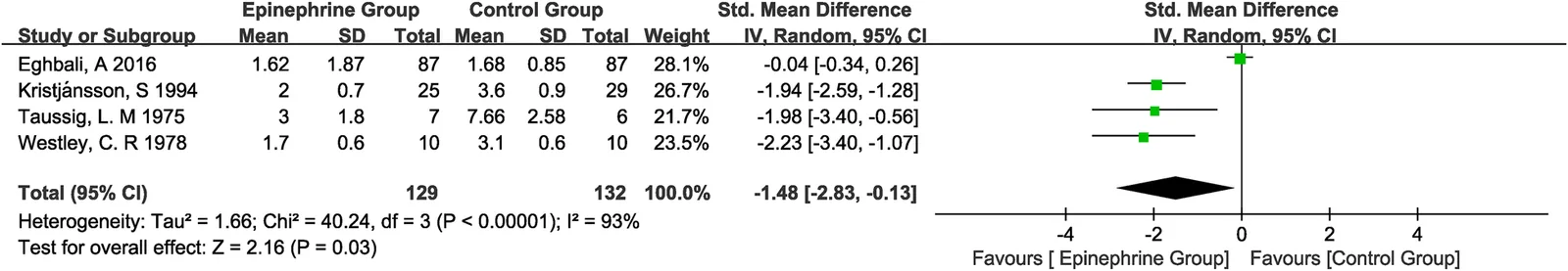
Comparison of comprehensive scores between epinephrine group and control group at 30 min after treatment for croup.

At 30 minuts, the pooled analysis showed a significant reduction in croup scores in the epinephrine group compared with the control group (SMD = −1.48, 95% CI [−2.83, −0.13], *P* = 0.03 < 0.05) (Figure 4). With substantial heterogeneity (I² = 93% > 50%, *P* < 0.1). This magnitude of improvement translates to a perceptible alleviation of laryngitis symptoms (e.g., stridor and respiratory distress) that can be readily observed at the bedside by attending physicians.

**Figure 5:**
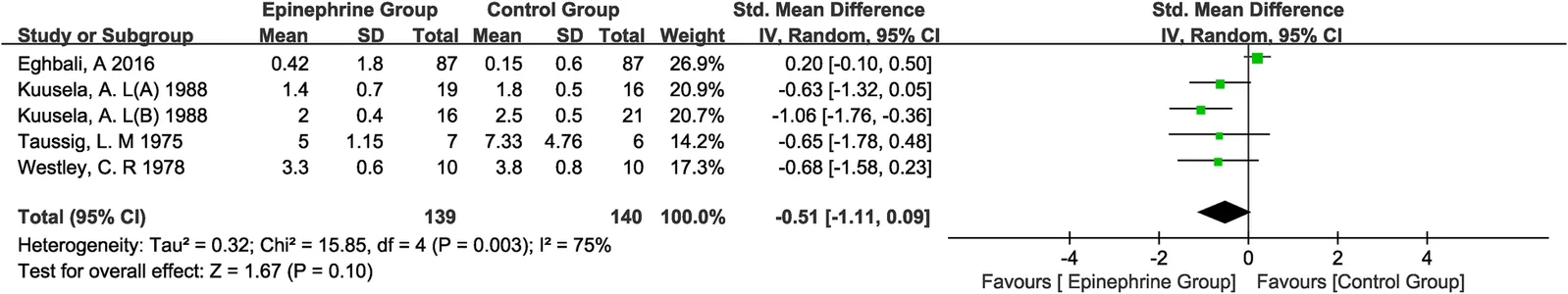
Comparison of comprehensive scores between epinephrine group and control group at 2 h after treatment for croup.

At 120 min after administration, the between-group difference in croup scores was no longer statistically significan (SMD = −0.51, 95% CI [−1.11, 0.09], *P* = 0.1), with considerable heterogeneity (I^2^ = 75% >50%, *P* = 0.003) (Figure 5).

Leave-one-out sensitivity analysis identified the study by Eghbali et al. (11) as the primary source of heterogeneity at both time points.

At 30 min, Leave-one-out exclusion of this study (11) reduced the I² from 93% to 0% and narrowed the confidence interval (SMD changed from −1.48, 95% CI [−2.83, −0.13] to −2.0, 95% CI [−2.54, −1.47]) (Supplementary Figure 1). Importantly, the 95% CI remained entirely below zero and the statistical significance was maintained, confirming that the 30 min effect is robust and not dependent on this single study. Eghbali et al (11). was the only study among these four in which both groups received dexamethasone as background therapy, a co-intervention that may have confounded the effect assessment and contributed to its outlying estimate.

At 120 min, however, exclusion of the same study (11) reversed the statistical conclusion: the pooled estimate changed from non-significant (SMD = −0.51, 95% CI [−1.11, 0.09], *P* = 0.1) to significant (SMD = −0.79, 95% CI [−1.19, −0.38], *P* < 0.05) (Supplementary Figure 2), indicating that the 120 min result is highly sensitive to the inclusion of this single study. Notably, the absolute scores in both groups at 120 min were substantially lower than at 30 min, which inherently limited the window for detecting a between-group difference. Another included study (21) also used dexamethasone as background therapy at 120 min, yet it did not introduce heterogeneity, suggesting that the influence of Eghbali et al. (11) was not simply explained by the concomitant use of dexamethasone. Other factors specific to Eghbali et al. (11), such as variations in epinephrine formulation, dosage, or timing of outcome assessment, may have contributed to its outlying estimate. Due to limited reporting in the original publications, we could not further validate these hypotheses through subgroup analyses. Taken together, the 120 min result is statistically fragile and does not permit a firm conclusion regarding the presence or absence of a sustained effect beyond 30 min. In contrast, the 30 min benefit of epinephrine appears robust and clinically meaningful.

#### Hospital stay

**Figure 6:**
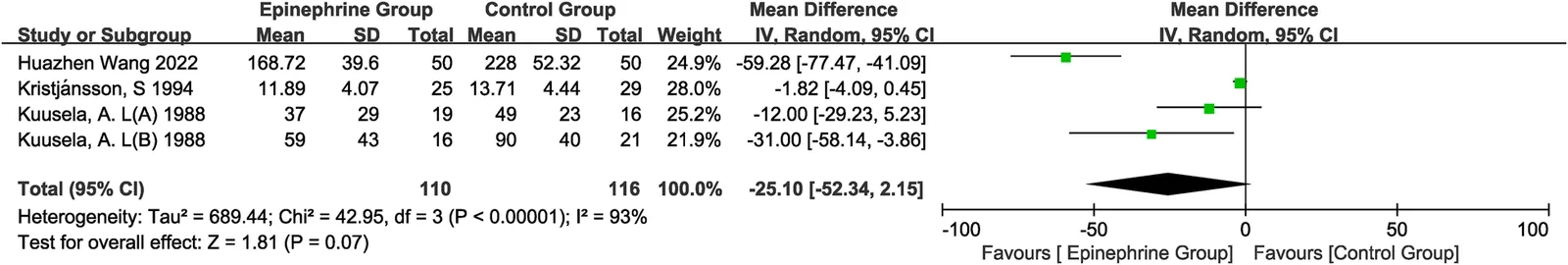
Comparison of hospital stay between epinephrine group and control group.

To ensure uniformity across studies, we converted all hospital stay durations to hours (e.g., multiplying days by 24) before pooling. The MD was used as the summary effect measure for this continuous outcome, with pooled estimates reported in hours. Three studies (18, 20, 21) reported on the length of hospital stay. The pooled analysis showed substantial heterogeneity (I^2^ = 93% >50%, *P* < 0.1), and a random-effects model was used. The pooled MD was—25.1 h (95% CI [−52.34, 2.15]) (*P* = 0.07 > 0.05) (Figure 6), which was not statistically significant between the epinephrine and control groups.

To explore the potential source of the substantial heterogeneity, we performed a leave-one-out sensitivity analysis by sequentially excluding each included study. Although the study by Huazhen Wang et al (18) (Supplementary Figure 3). was identified as the major contributor to the observed heterogeneity, the pooled effect estimates remained statistically nonsignificant across exclusion iterations, with the 95% confidence intervals consistently crossing zero. These findings demonstrated that the overall conclusion of no significant difference in hospital stay between the epinephrine and control groups was not dependent on any single study and remained robust despite the high heterogeneity.

### Publication bias

Because the number of original studies for each outcome indicator was less than 10, funnel plots and Egger's tests were not performed. To reduce the risk of bias, the following strategies were adopted in this study: (1) strict inclusion of only RCTs; (2) standardized risk of bias assessment; (3) sensitivity analysis were performed using Stata with a leave-one-out approach to assess the robustness of the pooled results.

### Narrative synthesis of studies not included in the meta-analysis

Among the 12 included studies (11–22), 6 studies (11, 17, 18, 20–22) provided sufficient continuous data (means and standard deviations) for the outcome and were subsequently pooled in the meta-analysis. The remaining 6 studies (12–16, 19) could not be quantitatively synthesized due to the absence of extractable numerical data. Their findings are summarized narratively below.

Duman et al. (15) compared three regimens in children with moderate to severe croup: cool mist with dexamethasone, nebulized L-epinephrine with dexamethasone, and nebulized L-epinephrine with budesonide. Epinephrine-containing groups showed faster improvement at 30 and 60 min vs. the cool mist group (*p* < 0.05). However, no differences were observed among groups at 90 or 120 min (*p* > 0.05). The results were presented only in graphical format without extractable means and standard deviations.

Weber et al. (12) compared heliox with nebulized epinephrine and reported comparable therapeutic efficacy between the two interventions in acute croup management. The results were presented only in graphical format without extractable means and standard deviations.

Fitzgerald et al. (16) compared nebulized L-epinephrine with nebulized budesonide for the treatment of croup and found no significant difference between the two groups. The results were reported in figure format with only *p* values provided, and no extractable means or standard deviations were available for meta-analysis.

Waisman et al. (13) directly compared L-epinephrine with racemic epinephrine and found that both agents produced temporary clinical improvement lasting 60–90 min, with no sustained superiority of either formulation.

Corkey et al. (14) compared nebulized distilled water with racemic epinephrine, both delivered via manual intermittent positive pressure ventilation, and found that epinephrine was significantly more effective than distilled water in providing acute relief of upper airway obstruction (*p* < 0.005). The study reported only *p* values, without providing extractable means or standard deviations.

Gardner et al. (19) conducted a double-blind, placebo-controlled trial in 20 children with infectious croup, comparing nebulized racemic epinephrine with saline placebo. The authors found that 5/10 (50%) patients in each group achieved significant clinical improvement (defined as a reduction in score ≥2). Only responder counts were reported, without means or standard deviations, precluding quantitative pooling.

Overall, the direction of effect across these six narratively synthesized studies was variable. Corkey et al. (14) reported a significant benefit of epinephrine over distilled water. Gardner et al. (19) found no difference between epinephrine and placebo. Fitzgerald et al. (16) demonstrated comparable efficacy between L-epinephrine and budesonide. Weber et al. (12) reported equivalent effects between heliox and epinephrine. Waisman et al. (13) showed no difference between the two epinephrine isomers. And Duman et al. (15) reported rapid improvement in epinephrine-containing groups, although data could not be pooled due to reporting format. Collectively, these narrative findings reveal no consistent pattern of effect across the non-pooled studies, ranging from significant benefit to no difference, and further reinforce the clinical and methodological diversity within the existing croup literature.

## Discussion

Croup is a common cause of upper airway obstruction in children and is characterised by hoarseness, a barking cough, and inspiratory stridor (1). Glucocorticoids are commonly used for controlling laryngeal edema (1). However, their onset of action is not immediate and typically takes 2 to 6 h (7, 25). For moderate to severe croup, nebulized epinephrine rapidly relieves airway obstruction (8, 22).

### The mechanism of action and pharmacological characteristics

Epinephrine is thought to improve symptoms in patients with croup through arteriole vasoconstriction in the upper airway mucosa, which eventually leads to decreased edema (26, 27). Epinephrine is typically used in conjunction with glucocorticoids because it has a quick onset of action but a short half-life (17, 28), whereas glucocorticoids have a slower onset of action but a longer half-life (3). Epinephrine plays a critical role in acute-phase management by rapidly alleviating laryngeal edema, thereby creating a time window for the slower onset of action of glucocorticoids and consequently reducing the rates of intubation and tracheostomy.

### Treatment outcome analysis

In this study, nebulized epinephrine demonstrated a significant and robust improvement in croup severity scores at 30 min post-administration. The magnitude of this effect (SMD = −1.48) was clinically substantial, corresponding to a perceptible alleviation of stridor and respiratory distress that could be readily recognized by attending physicians at the bedside. Importantly, this early benefit remained stable across leave-one-out sensitivity analyses, confirming that the finding was not driven by any single study. This robustness strengthens the credibility of the 30 min result and supports its generalizability across different clinical settings.

However, at 120 min, the between-group difference was no longer statistically significant. It is noteworthy that this null finding proved to be highly sensitive to the inclusion of a single study (11), as its exclusion reversed the statistical conclusion from non-significant to significant. This fragility must be considered when undertaking any definitive interpretation of the 120 min result. In view of the foregoing, the 120 min result is to be interpreted with caution: it does not permit a firm conclusion regarding the presence or absence of a sustained effect beyond 30 min. The paucity of reporting in the original publications precluded further subgroup analyses to explore the sources of this fragility, underscoring the need for future studies with standardised protocols and longer follow-up to clarify the trajectory of epinephrine's effect beyond the acute phase.

With regard to the duration of hospitalisation, the pooled analysis demonstrated a mean difference of −25.1 h in favour of the epinephrine group, although this did not attain statistical significance (95% CI [−52.34, 2.15]), *P* = 0.07). The direction and magnitude of this point estimate are of clinical interest; a potential reduction of approximately one day in hospitalization would carry considerable implications for both patient care and healthcare resource utilisation. However, the wide confidence interval crossing zero indicates that the estimate is imprecise, and the substantial heterogeneity across the included studies (18, 20, 21) (I^2^ = 93%) further complicates interpretation. Leave-one-out sensitivity analyses confirmed that the overall conclusion of no significant difference remained unchanged regardless of which study was excluded, suggesting that this negative finding was not dependent on any single study. The elevated heterogeneity, in conjunction with the limited number of studies, necessitates a cautious interpretation of the outcomes. Consequently, the observed trend is regarded as hypothesis-generating rather than confirmatory. It is recommended that future research be conducted with larger sample sizes and standardised discharge criteria to determine whether epinephrine truly confers a benefit in reducing hospitalization duration.

### Epinephrine in the treatment of croup: drug type, dosage, and delivery method

L-epinephrine is also more readily available worldwide, is less expensive. In a single study, Waisman et al. (13) reported that L-epinephrine and racemic epinephrine had comparable efficacy in the treatment of croup. There is currently no consensus on the optimal dosage. For 2.25% racemic epinephrine, the conventional dose is 0.05 mL/kg (maximum 0.5 mL) (1, 3). For 1:1000 L-epinephrine, the conventional dose is 0.5 mL/kg (maximum 5 mL) (3). Lee et al. (24) reported that low-dose epinephrine had comparable efficacy to conventional dosing. Fogel et al. (29) reported that comparable efficacy between nebulization alone and nebulization with intermittent positive pressure breathing (IPPB). Nebulization alone is better tolerated, simpler, less expensive, and avoids the potential risk of pneumothorax associated with IPPB. However, the findings regarding epinephrine type, dosage, and delivery method were each derived from single studies and were not systematically evaluated in our meta-analysis.

### Clinical monitoring and risk prevention

Although there are no reports of serious or obvious side effects at present, all children undergoing treatment require close monitoring. The reason is that nebulized epinephrine provides only temporary improvement of clinical symptoms due to its short duration of action.

### Limitations of the study

First, only RCTs were included; cohort studies, case-control studies, and other observational designs were excluded, resulting in a narrow range of study types. Second, the sample sizes of the included studies were relatively small, which may not adequately represent the broader patient population. Third, significant clinical heterogeneity existed among the included studies, as control groups were inconsistent and treatment regimens varied in both drug type and dosage. However, the limited number of included studies precluded meaningful subgroup analyses. Fourth, fewer than 10 studies contributed to each of the primary and secondary outcomes, precluding funnel plot analysis and Egger's test for publication bias. Fifth, We did't conduct an independent search of grey literature beyond trial registries; however, conference abstracts are partially indexed in the databases we searched. Nonetheless, some unpublished studies may have been missed. Sixth, our supplementary searches of ClinicalTrials.gov and WHO ICTRP identified three registered but unpublished RCTs that met our eligibility criteria (IRCT20241229064209N1, IRCT201105156491N1, IRCT201105264396N2). Their absence suggests potential publication bias, meaning our pooled effect estimates may be overestimated. Readers should interpret our findings with caution, and future updates should incorporate these trials once their results become available. Seventh, the timing of outcome assessment was inconsistent across studies, ranging from 30 min to 2 h, with no data at the 1 h time point. Additionally, clinical scores beyond 2 h were not reported, limiting the assessment of longer-term efficacy.

## Conclusion

Nebulized epinephrine remains a critical emergency treatment for upper airway obstruction caused by moderate-to-severe croup in children. This treatment rapidly reduces airway edema, as reflected by a significant improvement in croup score at 30 min post-administration, thereby allowing sufficient time for glucocorticoids to take effect. However, at 120 min, the pooled estimate was not statistically significant and was sensitive to the exclusion of a single study, indicating inconclusive evidence for sustained efficacy at 2 h. Close monitoring following epinephrine administration remains warranted. Regarding hospital stay, although the point estimate suggested a clinically meaningful reduction (approximately 25 h), the wide confidence interval crossed zero, precluding a definitive conclusion. The lack of statistical significance (*P* = 0.07) may reflect insufficient statistical power or heterogeneity in practice patterns across studies. Therefore, based on the current evidence, nebulized epinephrine should not be routinely recommended for the purpose of shortening hospital stay, although it retains an important role in acute emergency management when clinically indicated.

## Data Availability

The original contributions presented in the study are included in the article/Supplementary Material, further inquiries can be directed to the corresponding author/s.

## References

[1] Bjornson CL Johnson DW. Croup. Lancet. (2008) 371(9609):329–39. 10.1016/s0140-6736(08)60170-1 18295000 PMC7138055

[2] Denny FW Murphy TF Clyde WA Collier AM Henderson FW Senior RS. Croup: an 11-year study in a pediatric practice. Pediatrics. (1983) 71(6):871–6. 10.1542/peds.71.6.871 6304611

[3] Smith DK McDermott AJ Sullivan JF. Croup: diagnosis and management. Am Fam Physician. (2018) 97(9):575–80. 29763253

[4] Marx A Török TJ Holman RC Clarke MJ Anderson LJ. Pediatric hospitalizations for croup (laryngotracheobronchitis): biennial increases associated with human parainfluenza virus 1 epidemics. J Infect Dis. (1997) 176(6):1423–7. 10.1086/514137 9395350

[5] Aregbesola A Tam CM Kothari A Le ML Ragheb M Klassen TP. Glucocorticoids for croup in children. Cochrane Database Syst Rev. (2023) 2023(1):CD001955. 10.1002/14651858.CD001955.pub5 PMC9831289 36626194

[6] Klassen TP Craig WR Moher D Osmond MH Pasterkamp H Sutcliffe T. Nebulized budesonide and oral dexamethasone for treatment of croup: a randomized controlled trial. Jama. (1998) 279(20):1629–32. 10.1001/jama.279.20.1629 9613912

[7] Ausejo M Saenz A Pham B Kellner JD Johnson DW Moher D. The effectiveness of glucocorticoids in treating croup: meta-analysis. Br Med J. (1999) 319(7210):595–600. 10.1136/bmj.319.7210.595 10473471 PMC28209

[8] Singer OP Wilson WJ. Laryngotracheobronchitis: 2 Years’ experience with racemic epinephrine. Can Med Assoc J. (1976) 115(2):132–4. 776385 PMC1878550

[9] Rizos JD DiGravio BE Sehl MJ Tallon JM. The disposition of children with croup treated with racemic epinephrine and dexamethasone in the emergency department. J Emerg Med. (1998) 16(4):535–9. 10.1016/s0736-4679(98)00055-9 9696166

[10] Page MJ McKenzie JE Bossuyt PM Boutron I Hoffmann TC Mulrow CD. The prisma 2020 statement: an updated guideline for reporting systematic reviews. Br Med J. (2021) 372:n71. 10.1136/bmj.n71 33782057 PMC8005924

[11] Eghbali A Sabbagh A Bagheri B Taherahmadi H Kahbazi M. Efficacy of nebulized L-epinephrine for treatment of croup: a randomized, double-blind study. Fundam Clin Pharmacol. (2016) 30(1):70–5. 10.1111/fcp.12158 26463007

[12] Weber JE Chudnofsky CR Younger JG Larkin GL Boczar M Wilkerson MD. A randomized comparison of helium-oxygen mixture (heliox) and racemic epinephrine for the treatment of moderate to severe croup. Pediatrics. (2001) 107(6):E96–E96. 10.1542/peds.107.6.e96 11389294

[13] Waisman Y Klein BL Boenning DA Young GM Chamberlain JM O'Donnell R. Prospective randomized double-blind study comparing L-epinephrine and racemic epinephrine aerosols in the treatment of laryngotracheitis (croup). Pediatrics. (1992) 89(2):302–6. 10.1542/peds.89.2.302 1734400

[14] Corkey CW Barker GA Edmonds JF Mok PM Newth CJ. Radiographic tracheal diameter measurements in acute infectious croup: an objective scoring system. Crit Care Med. (1981) 9(8):587–90. 10.1097/00003246-198108000-00007 7021067

[15] Duman M Özdemir D Atasever S. Nebulised L-epinephrine and steroid combination in the treatment of moderate to severe croup. Clin Drug Investig. (2005) 25(3):183–9. 10.2165/00044011-200525030-00004 17523767

[16] Fitzgerald D Mellis C Johnson M Allen H Cooper P Van Asperen P. Nebulized budesonide is as effective as nebulized adrenaline in moderately severe croup. Pediatrics. (1996) 97(5):722–5. 10.1203/00006450-199605000-00028 8628614

[17] Taussig LM Castro O Beaudry PH Fox WW Bureau M. Treatment of laryngotracheobronchitis (croup). use of intermittent positive-pressure breathing and racemic epinephrine. Am J Dis Child. (1975) 129(7):790–3. 10.1001/archpedi.1975.02120440016004 1096594

[18] Wang HZ Han NQ Xu NN Wu QJ Dong XQ Han Z. Efficacy of combined nebulized aerosol inhalation and pulmicort respules in the treatment of emergency pediatric laryngitis, and its effect on adverse reactions. Trop J Pharm Res. (2022) 21(6):1317–22. 10.4314/tjpr.v21i6.26

[19] Gardner HG Powell KR Roden VJ Cherry JD. The evaluation of racemic epinephrine in the treatment of infectious croup. Pediatrics. (1973) 52(1):52–5. 10.1542/peds.52.1.52 4579587

[20] Kristjánsson S Berg-Kelly K Winsö E. Inhalation of racemic Adrenaline in the treatment of mild and moderately severe croup. Clinical symptom score and oxygen saturation measurements for evaluation of treatment effects. Acta Paediatr. (1994) 83(11):1156–60. 10.1111/j.1651-2227.1994.tb18270.x 7841729

[21] Kuusela AL Vesikari T. A randomized double-blind, placebo-controlled trial of dexamethasone and racemic epinephrine in the treatment of croup. Acta Paediatr Scand. (1988) 77(1):99–104. 10.1111/j.1651-2227.1988.tb10606.x 3285638

[22] Westley CR Cotton EK Brooks JG. Nebulized racemic epinephrine by ippb for the treatment of croup: a double-blind study. Am J Dis Child. (1978) 132(5):484–7. 10.1001/archpedi.1978.02120300044008 347921

[23] Bjornson C Russell KF Vandermeer B Durec T Klassen TP Johnson DW. Nebulized epinephrine for croup in children. Cochrane Database Syst Rev. (2011) 2:CD006619. 10.1002/14651858.CD006619.pub2 21328284

[24] Lee JH Jung JY Lee HJ Kim DK Kwak YH Chang I. Efficacy of low-dose nebulized epinephrine as treatment for croup: a randomized, placebo-controlled, double-blind trial. Am J Emerg Med. (2019) 37(12):2171–6. 10.1016/j.ajem.2019.03.012 30878411

[25] Gates A Johnson DW Klassen TP. Glucocorticoids for croup in Children. JAMA Pediatr. (2019) 173(6):595–6. 10.1001/jamapediatrics.2019.0834 31033996

[26] Hou YT Shi Q Zhang L Cheng Q. Clinical advances in racemic epinephrine for pediatric croup: a Mini-review of evidence and practice. Front Pediatr. (2025) 13:1616521. 10.3389/fped.2025.1616521 40625385 PMC12230069

[27] Leung K Newth CJL Hotz JC O'Brien KC Fink JB Coates AL. Delivery of epinephrine in the vapor phase for the treatment of croup. Pediatr Crit Care Med. (2016) 17(4):E177–E81. 10.1097/pcc.0000000000000666 26910479

[28] Pineda Celis A Valle Farías JL Juárez Aragón G Games Eternod J Serafín FJ. Evaluation of racemic epinephrine with intermittent positive pressure in the treatment of acute infections laryngotracheitis. Bol Med Hosp Infant Mex. (1978) 35(4):599–607. 348210

[29] Fogel JM Berg IJ Gerber MA Sherter CB. Racemic epinephrine in the treatment of croup: nebulization alone versus nebulization with intermittent positive pressure breathing. J Pediatr. (1982) 101(6):1028–31. 10.1016/s0022-3476(82)80039-5 6754899

