# Supplementary material for: Nebulized epinephrine for croup in children: an updated systematic review
Source: Front Pediatr. 2026 Aug 26;14:1886410. doi: 10.3389/fped.2026.1886410 (PMC13553941; doi:10.3389/fped.2026.1886410)
Supplement: Supplementary file 1 [file Table1.docx]

**Appendix Table 1** Search strategies for PubMed (MEDLINE), Embase, Scopus, the Cochrane Library and Web of science

| **No.** | **Search terms** | Results |
| --- | --- | --- |
| **Pubmed (MEDLINE) (Search date：July 3, 2026)** | | |
| #1 | "Croup"[Mesh] | 1277 |
| #2 | (Viral Croup[Title/Abstract]) OR (Croup, Viral[Title/Abstract]) | 332 |
| #3  (#1 OR #2) | ("Croup"[Mesh]) OR ((Viral Croup[Title/Abstract]) OR (Croup, Viral[Title/Abstract])) | 1430 |
| #4 | Laryngitis"[Mesh] | 4210 |
| #5 | (Laryngitides[Title/Abstract]) OR (laryngotracheitis[Title/Abstract]) | 1085 |
| #6  (#4 OR #5) | ("Laryngitis"[Mesh]) OR ((Laryngitides[Title/Abstract]) OR (laryngotracheitis[Title/Abstract])) | 5079 |
| #7  (#3 OR #6) | (("Croup"[Mesh]) OR ((Viral Croup[Title/Abstract]) OR (Croup, Viral[Title/Abstract]))) OR (("Laryngitis"[Mesh]) OR ((Laryngitides[Title/Abstract]) OR (laryngotracheitis[Title/Abstract]))) | 5220 |
| #8 | "Epinephrine"[Mesh] | 58410 |
| #9 | ((((((((((((((((racemic epinephrine[Title/Abstract]) OR (Adrenaline[Title/Abstract])) OR (Epinephrine Bitartrate[Title/Abstract])) OR (Epinephrine Hydrochloride[Title/Abstract])) OR (Adrenaline Bitartrate[Title/Abstract])) OR (Adrenaline Hydrochloride[Title/Abstract])) OR (adrenalin*[Title/Abstract])) OR (l‐adrenalin[Title/Abstract])) OR (l‐adrenaline[Title/Abstract])) OR (epinephrin*[Title/Abstract])) OR (adrenergic agonist[Title/Abstract])) OR (adrenergic agonists[Title/Abstract])) OR (adrenergic alpha‐agonist[Title/Abstract])) OR (adrenergic alpha‐agonists[Title/Abstract])) OR (adrenergic beta‐agonist[Title/Abstract])) OR (adrenergic beta‐agonists[Title/Abstract])) OR (adrenergic receptor stimulating agent[Title/Abstract]) | 74841 |
| #10  (#8 OR #9) | ("Epinephrine"[Mesh]) OR (((((((((((((((((racemic epinephrine[Title/Abstract]) OR (Adrenaline[Title/Abstract])) OR (Epinephrine Bitartrate[Title/Abstract])) OR (Epinephrine Hydrochloride[Title/Abstract])) OR (Adrenaline Bitartrate[Title/Abstract])) OR (Adrenaline Hydrochloride[Title/Abstract])) OR (adrenalin*[Title/Abstract])) OR (l‐adrenalin[Title/Abstract])) OR (l‐adrenaline[Title/Abstract])) OR (epinephrin*[Title/Abstract])) OR (adrenergic agonist[Title/Abstract])) OR (adrenergic agonists[Title/Abstract])) OR (adrenergic alpha‐agonist[Title/Abstract])) OR (adrenergic alpha‐agonists[Title/Abstract])) OR (adrenergic beta‐agonist[Title/Abstract])) OR (adrenergic beta‐agonists[Title/Abstract])) OR (adrenergic receptor stimulating agent[Title/Abstract])) | 95909 |
| #11  (#7 AND #10) | **(English[Language]) AND (((("Croup"[Mesh]) OR ((Viral Croup[Title/Abstract]) OR (Croup, Viral[Title/Abstract]))) OR (("Laryngitis"[Mesh]) OR ((Laryngitides[Title/Abstract]) OR (laryngotracheitis[Title/Abstract])))) AND (("Epinephrine"[Mesh]) OR (((((((((((((((((racemic epinephrine[Title/Abstract]) OR (Adrenaline[Title/Abstract])) OR (Epinephrine Bitartrate[Title/Abstract])) OR (Epinephrine Hydrochloride[Title/Abstract])) OR (Adrenaline Bitartrate[Title/Abstract])) OR (Adrenaline Hydrochloride[Title/Abstract])) OR (adrenalin*[Title/Abstract])) OR (l‐adrenalin[Title/Abstract])) OR (l‐adrenaline[Title/Abstract])) OR (epinephrin*[Title/Abstract])) OR (adrenergic agonist[Title/Abstract])) OR (adrenergic agonists[Title/Abstract])) OR (adrenergic alpha‐agonist[Title/Abstract])) OR (adrenergic alpha‐agonists[Title/Abstract])) OR (adrenergic beta‐agonist[Title/Abstract])) OR (adrenergic beta‐agonists[Title/Abstract])) OR (adrenergic receptor stimulating agent[Title/Abstract]))))** | 242 |
|  | | |
| **Embase (Search date：July 3, 2026)** | | |
| #1 | 'epinephrine'/exp | 155231 |
| #2 | 'racemic epinephrine':ab,ti OR 'adrenaline':ab,ti OR 'epinephrine bitartrate':ab,ti OR 'epinephrine hydrochloride':ab,ti OR 'adrenergic agonists':ab,ti OR 'adrenergic alpha‐agonist':ab,ti OR 'adrenergic alpha‐agonists':ab,ti OR 'adrenergic beta‐agonist':ab,ti OR 'adrenergic beta‐agonists':ab,ti OR 'adrenaline bitartrate':ab,ti OR 'adrenaline hydrochloride':ab,ti OR 'adrenalin*':ab,ti OR 'l‐adrenalin':ab,ti OR 'l‐adrenaline':ab,ti OR 'epinephrin*':ab,ti OR 'adrenergic agonist':ab,ti | 98294 |
| #3 (#1 OR #2) | 'epinephrine'/exp OR 'racemic epinephrine':ab,ti OR 'adrenaline':ab,ti OR 'epinephrine bitartrate':ab,ti OR 'epinephrine hydrochloride':ab,ti OR 'adrenergic agonists':ab,ti OR 'adrenergic alpha‐agonist':ab,ti OR 'adrenergic alpha‐agonists':ab,ti OR 'adrenergic beta‐agonist':ab,ti OR 'adrenergic beta‐agonists':ab,ti OR 'adrenaline bitartrate':ab,ti OR 'adrenaline hydrochloride':ab,ti OR 'adrenalin*':ab,ti OR 'l‐adrenalin':ab,ti OR 'l‐adrenaline':ab,ti OR 'epinephrin*':ab,ti OR 'adrenergic agonist':ab,ti | 18323 |
| #4 | 'laryngitis'/exp | 9294 |
| #5 | 'croup':ab,ti OR 'viral croup':ab,ti OR 'croup, viral':ab,ti OR 'laryngitides':ab,ti OR 'laryngotracheitis':ab,ti | 3503 |
| #6 #4 OR #5 | 'laryngitis'/exp OR 'croup':ab,ti OR 'viral croup':ab,ti OR 'croup, viral':ab,ti OR 'laryngitides':ab,ti OR 'laryngotracheitis':ab,ti | 10661 |
| #7 #3 AND #6 | ('epinephrine'/exp OR 'racemic epinephrine':ab,ti OR 'adrenaline':ab,ti OR 'epinephrine bitartrate':ab,ti OR 'epinephrine hydrochloride':ab,ti OR 'adrenergic agonists':ab,ti OR 'adrenergic alpha‐agonist':ab,ti OR 'adrenergic alpha‐agonists':ab,ti OR 'adrenergic beta‐agonist':ab,ti OR 'adrenergic beta‐agonists':ab,ti OR 'adrenaline bitartrate':ab,ti OR 'adrenaline hydrochloride':ab,ti OR 'adrenalin*':ab,ti OR 'l‐adrenalin':ab,ti OR 'l‐adrenaline':ab,ti OR 'epinephrin*':ab,ti OR 'adrenergic agonist':ab,ti) AND ('laryngitis'/exp OR 'croup':ab,ti OR 'viral croup':ab,ti OR 'croup, viral':ab,ti OR 'laryngitides':ab,ti OR 'laryngotracheitis':ab,ti) | 861 |
| #8 #7 AND english:la | **('epinephrine'/exp OR 'racemic epinephrine':ab,ti OR 'adrenaline':ab,ti OR 'epinephrine bitartrate':ab,ti OR 'epinephrine hydrochloride':ab,ti OR 'adrenergic agonists':ab,ti OR 'adrenergic alpha‐agonist':ab,ti OR 'adrenergic alpha‐agonists':ab,ti OR 'adrenergic beta‐agonist':ab,ti OR 'adrenergic beta‐agonists':ab,ti OR 'adrenaline bitartrate':ab,ti OR 'adrenaline hydrochloride':ab,ti OR 'adrenalin*':ab,ti OR 'l‐adrenalin':ab,ti OR 'l‐adrenaline':ab,ti OR 'epinephrin*':ab,ti OR 'adrenergic agonist':ab,ti) AND ('laryngitis'/exp OR 'croup':ab,ti OR 'viral croup':ab,ti OR 'croup, viral':ab,ti OR 'laryngitides':ab,ti OR 'laryngotracheitis':ab,ti) AND english:la** | 732 |
| **Cochrane Library (Search date：July 3, 2026)** | | |
| #1 | MeSH descriptor: [Epinephrine] explode all trees | 4431 |
| #2 | (racemic epinephrine):ab,ti,kw OR (Adrenaline):ab,ti,kw OR (Epinephrine Bitartrate):ab,ti,kw OR (Epinephrine Hydrochloride):ab,ti,kw OR (Adrenaline Bitartrate):ab,ti,kw OR (Adrenaline Hydrochloride):ab,ti,kw OR (adrenalin*):ab,ti,kw OR (l‐adrenalin):ab,ti,kw OR (l‐adrenaline):ab,ti,kw | 5457 |
| #3 | #1 OR #2 | 8381 |
| #4 | MeSH descriptor: [Croup] explode all trees | 92 |
| #5 | (Viral Croup):ab,ti,kw OR (Croup, Viral):ab,ti,kw OR (Laryngitides):ab,ti,kw OR (laryngotracheitis):ab,ti,kw OR (Laryngitis):ab,ti,kw | 321 |
| #6 | #4 OR #5 | 388 |
| #7 | #3 AND #6 | 46 |
|  |  |  |
| **Web of science (Search date：July 3, 2026)** | | |
| #1 | TS=(Laryngitis OR Laryngitides OR Croup OR Viral Croup OR Croup, Viral OR laryngotracheitis) | 4582 |
| #2 | TS=(Epinephrine OR racemic epinephrine OR Adrenaline OR Epinephrine Bitartrate OR Epinephrine Hydrochloride OR Adrenaline Bitartrate OR Adrenaline Hydrochloride OR adrenalin* OR l‐adrenalin OR l‐adrenaline OR epinephrin* OR adrenergic agonist OR adrenergic agonists OR adrenergic alpha‐agonist OR adrenergic alpha‐agonists OR adrenergic beta‐agonist OR adrenergic beta‐agonists OR adrenergic receptor stimulating agent) | 77407 |
| #3 | TS=(Laryngitis OR Laryngitides OR Croup OR Viral Croup OR Croup, Viral OR laryngotracheitis) AND TS=(Epinephrine OR racemic epinephrine OR Adrenaline OR Epinephrine Bitartrate OR Epinephrine Hydrochloride OR Adrenaline Bitartrate OR Adrenaline Hydrochloride OR adrenalin* OR l‐adrenalin OR l‐adrenaline OR epinephrin* OR adrenergic agonist OR adrenergic agonists OR adrenergic alpha‐agonist OR adrenergic alpha‐agonists OR adrenergic beta‐agonist OR adrenergic beta‐agonists OR adrenergic receptor stimulating agent) | 239 |
| #4 | **((TS=((Laryngitis OR Laryngitides OR Croup OR Viral Croup OR Croup, Viral OR laryngotracheitis))) AND TS=((Epinephrine OR racemic epinephrine OR Adrenaline OR Epinephrine Bitartrate OR Epinephrine Hydrochloride OR Adrenaline Bitartrate OR Adrenaline Hydrochloride OR adrenalin* OR l‐adrenalin OR l‐adrenaline OR epinephrin* OR adrenergic agonist OR adrenergic agonists OR adrenergic alpha‐agonist OR adrenergic alpha‐agonists OR adrenergic beta‐agonist OR adrenergic beta‐agonists OR adrenergic receptor stimulating agent) )) AND LA=(English)** | 225 |
|  | | |
| **Scopus (Search date：July 3, 2026)** | | |
| #1 | ( TITLE-ABS-KEY ( **"****Epinephrine" OR "Adrenaline" OR "racemic epinephrine" OR "Epinephrine Bitartrate" OR "Epinephrine Hydrochloride" OR "Adrenaline Bitartrate" OR "adrenalin*" OR "l‐adrenalin" OR "l‐adrenaline" OR "Adrenaline Hydrochloride" OR "epinephrin*" OR "adrenergic agonist" OR "adrenergic agonists" OR "adrenergic alpha‐agonist" OR "adrenergic alpha‐agonists" OR "adrenergic beta‐agonist" OR "adrenergic beta‐agonists" OR "adrenergic receptor stimulating agent"** ) AND TITLE-ABS-KEY ( **"Croup" OR "Viral Croup" OR "Croup, Viral" OR "Laryngitis" OR "Laryngitides" OR "laryngotracheitis"** ) AND **LANGUAGE ( English )** ) | 712 |
