# Supplementary material for: Nebulized epinephrine for croup in children: an updated systematic review
Source: Front Pediatr. 2026 Aug 26;14:1886410. doi: 10.3389/fped.2026.1886410 (PMC13553941; doi:10.3389/fped.2026.1886410)
Supplement: Supplementary file 2 [file Table2.docx]

**Appendix Table 2 Studies excluded and reasons for exclusion**

| **Study** | **Reasons for Exclusion** |
| --- | --- |
| *(Akcan Yildiz et al. 2024)* | Not a randomized controlled trial. |
| *(Armstrong et al. 2024)* |  |
| *(Asmundsson et al. 2019)* |  |
| *(Bagwell et al. 2020)* |  |
| *(Bass 1978)* |  |
| *(Caleb Hancock et al. 2023)* |  |
| *(Chiang et al. 2019)* |  |
| *(Chin et al. 2002)* |  |
| *(Choi et al. 2022)* |  |
| *(Elder & Rao 2019)* |  |
| *(Hancock et al. 2021)* |  |
| *(Hancock et al. 2023)* |  |
| *(Hano et al. 2025)* |  |
| *(Hey et al. 2025)* |  |
| *(Kahne et al. 2022)* |  |
| *(Kelley & Simon 1992)* |  |
| *(Kim et al. 2024a)* |  |
| *(Kim et al. 2024b)* |  |
| *(Kunkel & Baker 1996)* |  |
| *(Ledwith et al. 1995)* |  |
| *(McDonogh 1994)* |  |
| *(Mietens et al. 1984)* |  |
| *(Narayanan & Funkhouser 2014)* |  |
| *(Pierantoni et al. 2024)* |  |
| *(Postma et al. 1984)* |  |
| *(Pourjoula et al. 2025)* |  |
| *(Prendergast et al. 1994)* |  |
| *(Rizos et al. 1998)* |  |
| *(Rudinsky et al. 2015)* |  |
| *(Singer & Wilson 1976)* |  |
| *(Smith et al. 2018)* |  |
| *(Song et al. 2024)* |  |
| *(Tan et al. 2025)* |  |
| *(Walsh & Lipshaw 2022)* |  |
| *(Walsh et al. 2023)* |  |
| *(Winter et al. 2021)* |  |
| *(Wyly et al. 2025)* |  |
| *(Al Khaldi et al. 2002)* | Compares dexamethasone versus placebo. |
| *(Baranwal et al. 2021)* | This study investigates the use of dexamethasone for the prevention of post-extubation airway obstruction in children. |
| *(Fifoot & Ting 2007)* | To compare the effectiveness of three corticosteroid regimens in children with mild to moderate croup. |
| *(Geelhoed & Macdonald 1995)* | To compare the efficacy of oral dexamethasone and inhaled budesonide in children hospitalized with croup. |
| *(Marañón Pardillo et al. 2009)* | The objective of the study was to evaluate the efficacy of the administration of heliox in croup. |
| *(Neto et al. 2002)* | Children with croup were randomly assigned to receive either mist (humidified oxygen) via mist stick or no mist. |
| *(Preutthipan et al. 2005)* | The study enrolled children who developed croup with upper airway obstruction following tracheal extubation. |
| *(Ritu & Jhamb 2020)* |  |
| *(Scolnik et al. 2006)* | To compare the effect on clinical Westley croup scores among children with moderate to severe croup in the emergency department receiving either 100% humidity, 40% humidity, or traditional blow-by humidity. |
| *(Sumboonnanonda et al. 1997)* | Compares dexamethasone versus placebo. |
| *(Super et al. 1989)* | Compares dexamethasone versus placebo (saline). |
| *(Lee et al. 2019)* | The therapeutic effects of different doses of epinephrine in the treatment of croup. |
| *(Fogel et al. 1982)* | Treatment of croup with different administration methods of epinephrine. |
| *(Martínez Fernández et al. 1993)* | Not in English language. |

**Akcan Yildiz L, Akca H, Kurt F, Hanalioglu D, Cetin M, Senel S, and Karacan CD**. 2024. Improving croup management at a pediatric emergency department. *Postgraduate Medicine* **136**:438-445. <http://dx.doi.org/10.1080/00325481.2024.2360889>

**Al Khaldi O, Raggad M, and Aldhanhani MS**. 2002. Effect of dexamethasone in acute laryngotracheitis. *Qatar Medical Journal* **11**:44-46.

**Armstrong S, Bogle D, Carey K, and MacInnis M**. 2024. Evaluation of the Management of Croup in a Pediatric Emergency Department Before and After the COVID-19 Pandemic. *Canadian Journal of Hospital Pharmacy* **77**:8. <http://dx.doi.org/10.4212/cjhp.3607>

**Asmundsson AS, Arms J, Kaila R, Roback MG, Theiler C, Davey CS, and Louie JP**. 2019. Hospital Course of Croup After Emergency Department Management. *Hosp Pediatr* **9**:326-332. <http://dx.doi.org/10.1542/hpeds.2018-0066>

**Bagwell T, Hollingsworth A, Thompson T, Abramo T, Huckabee M, Chang D, Crawley L, and Robbins JM**. 2020. Management of Croup in the Emergency Department: The Role of Multidose Nebulized Epinephrine. *Pediatr Emerg Care* **36**:e387-e392. <http://dx.doi.org/10.1097/pec.0000000000001276>

**Baranwal A, Parajuli B, Kumar-M P, and Jayashree M**. 2021. 24-hour pretreatment with high dose (0.5mg/kg/dose) versus low dose (0.25mg/kg/dose) dexamethasone for prevention of post-extubation airway obstruction in children: A randomized open-label trial. *Pediatric Critical Care Medicine* **22**:21. <http://dx.doi.org/10.1097/01.pcc.0000738244.92607.cc>

**Bass JW**. 1978. Croup: IPPB and/or racemic epinephrine therapy. *Pediatrics* **61**:148-149.

**Caleb Hancock W, Scott M, and Winer JC**. 2023. Predictors of Inpatient Racemic Epinephrine Use in Patients Admitted With Croup. *Hospital Pediatrics* **13**:258-264. <http://dx.doi.org/10.1542/hpeds.2022-006870>

**Chiang E, Afandi O, Lee SH, Suresh S, Pitetti RD, and Ramgopal S**. 2019. Inpatient use of racemic epinephrine for children admitted with croup. *World J Pediatr* **15**:586-594. <http://dx.doi.org/10.1007/s12519-019-00291-x>

**Chin R, Browne GJ, Lam LT, McCaskill ME, Fasher B, and Hort J**. 2002. Effectiveness of a croup clinical pathway in the management of children with croup presenting to an emergency department. *J Paediatr Child Health* **38**:382-387. <http://dx.doi.org/10.1046/j.1440-1754.2002.00011.x>

**Choi YY, Kim YS, Lee SY, Sim J, Choe YJ, and Han MS**. 2022. Croup as a Manifestation of SARS-CoV-2 Omicron Variant Infection in Young Children. *J Korean Med Sci* **37**:e140. <http://dx.doi.org/10.3346/jkms.2022.37.e140>

**Elder AE, and Rao A**. 2019. Management and outcomes of patients presenting to the emergency department with croup: Can we identify which patients can safely be discharged from the emergency department? *J Paediatr Child Health* **55**:1323-1328. <http://dx.doi.org/10.1111/jpc.14412>

**Fifoot AA, and Ting JY**. 2007. Comparison between single-dose oral prednisolone and oral dexamethasone in the treatment of croup: a randomized, double-blinded clinical trial. *Emerg Med Australas* **19**:51-58. <http://dx.doi.org/10.1111/j.1742-6723.2006.00919.x>

**Fogel JM, Berg IJ, Gerber MA, and Sherter CB**. 1982. Racemic epinephrine in the treatment of croup: nebulization alone versus nebulization with intermittent positive pressure breathing. *J Pediatr* **101**:1028-1031. <http://dx.doi.org/10.1016/s0022-3476(82)80039-5>

**Geelhoed GC, and Macdonald WBG**. 1995. Oral and inhaled steroids in croup: A randomized, placebo‐controlled trial. *Pediatric pulmonology* **20**:355-361. <http://dx.doi.org/10.1002/ppul.1950200604>

**Hancock WC, Scott M, and Winer J**. 2021. Predicting inpatient croup outcomes. *Journal of Investigative Medicine* **69**:467-468. <http://dx.doi.org/10.1136/jim-2021-SRMC.125>

**Hancock WC, Scott M, and Winer JC**. 2023. Predictors of Inpatient Racemic Epinephrine Use in Patients Admitted With Croup. *Hosp Pediatr* **13**:258-264. <http://dx.doi.org/10.1542/hpeds.2022-006870>

**Hano D, Hametz P, and Warman K**. 2025. Risk Factors Associated With Emergency Department Revisits and Subsequent Hospitalizations in Previously Healthy Children Treated for Croup. *Hosp Pediatr* **15**:652-659. <http://dx.doi.org/10.1542/hpeds.2024-008093>

**Hey F, Lieftüchter V, Olivieri M, Zimatschek S, Hoffmann F, and Pfeiffer D**. 2025. Treatment pathways and rebound-rate of prehospital viral croup attacks-data from a prehospital pediatric physician led emergency service-a prospective observational follow-up study. *Front Pediatr* **13**:1544480. <http://dx.doi.org/10.3389/fped.2025.1544480>

**Kahne K, Krief W, Victoria P, and Dumont O**. 2022. 255 Delivery of Epinephrine by Metered-Dose Inhaler for the Treatment of Croup in Children. *Annals of Emergency Medicine* **80**:S111. <http://dx.doi.org/10.1016/j.annemergmed.2022.08.282>

**Kelley PB, and Simon JE**. 1992. Racemic epinephrine use in croup and disposition. *Am J Emerg Med* **10**:181-183. <http://dx.doi.org/10.1016/0735-6757(92)90204-b>

**Kim BH, Ha EK, Yoo H, Lee S, Rha YH, Han MY, and Kim JH**. 2024a. Trends in Incidence and Drug Prescriptions for Croup in Children Under 5 Years of Age: A 2002-2019 Population-Based Study. *J Korean Med Sci* **39**:e95. <http://dx.doi.org/10.3346/jkms.2024.39.e95>

**Kim M, Shin M, Lee Y, and Han S**. 2024b. Factors predicting hospitalization of pediatric patients with croup presenting to the emergency department: a retrospective cross-sectional study. *Signa Vitae* **20**:68-74. <http://dx.doi.org/10.22514/sv.2024.100>

**Kunkel NC, and Baker MD**. 1996. Use of racemic epinephrine, dexamethasone, and mist in the outpatient management of croup. *Pediatr Emerg Care* **12**:156-159. <http://dx.doi.org/10.1097/00006565-199606000-00003>

**Ledwith CA, Shea LM, and Mauro RD**. 1995. Safety and efficacy of nebulized racemic epinephrine in conjunction with oral dexamethasone and mist in the outpatient treatment of croup. *Ann Emerg Med* **25**:331-337. <http://dx.doi.org/10.1016/s0196-0644(95)70290-3>

**Lee JH, Jung JY, Lee HJ, Kim DK, Kwak YH, Chang I, Kwon H, Choi YJ, Park JW, Paek SH, and Cho JH**. 2019. Efficacy of low-dose nebulized epinephrine as treatment for croup: A randomized, placebo-controlled, double-blind trial. *Am J Emerg Med* **37**:2171-2176. <http://dx.doi.org/10.1016/j.ajem.2019.03.012>

**Marañón Pardillo R, Peñalba Citores A, Míguez Navarro C, Guerrero Soler M, and Vázquez López P**. 2009. Heliox as treatment for mild croup. *Acta Pediatrica Espanola* **67**:432-434.

**Martínez Fernández A, Sánchez González E, Rica Etxebarría I, Echaniz Urcelay I, Alonso Díez M, Vilella Ciriza M, and Gárate Aranzadi J**. 1993. Randomized double-blind study of treatment of croup with adrenaline and/or dexamethasone in children. *Anales espanoles de pediatria* **38**:29‐32.

**McDonogh AJ**. 1994. The use of steroids and nebulised adrenaline in the treatment of viral croup over a seven year period at a district hospital. *Anaesth Intensive Care* **22**:175-178. <http://dx.doi.org/10.1177/0310057x9402200210>

**Mietens C, Lutkemeyer B, and Kohler S**. 1984. Epidemiology and symptomatology of infectious croup. (Stenosing laryngotracheitis) in 1322 hospitalized patients in Bochum during the last 17 years. *Monatsschrift fur Kinderheilkunde* **132**:646-653.

**Narayanan S, and Funkhouser E**. 2014. Inpatient hospitalizations for croup. *Hosp Pediatr* **4**:88-92. <http://dx.doi.org/10.1542/hpeds.2013-0066>

**Neto GM, Kentab O, Klassen TP, and Osmond MH**. 2002. A randomized controlled trial of mist in the acute treatment of moderate croup. *Academic emergency medicine* **9**:873-879. <http://dx.doi.org/10.1197/aemj.9.9.873>

**Pierantoni L, Andreozzi L, Stera G, Toschi Vespasiani G, Biagi C, Zama D, Balduini E, Scheier LM, and Lanari M**. 2024. National survey conducted among Italian pediatricians examining the therapeutic management of croup. *Respir Med* **226**:107587. <http://dx.doi.org/10.1016/j.rmed.2024.107587>

**Postma DS, Jones RO, and Pillsbury Iii HC**. 1984. Severe hospitalized croup: Treatment trends and prognosis. *Laryngoscope* **94**:1170-1175.

**Pourjoula F, Mirlohi SH, and Ghanbari N**. 2025. Comparative analysis of croup severity and treatment in pediatric patient: a study of COVID-19 positive vs. negative cases during peak Omicron. *BMC Pediatr* **25**:194. <http://dx.doi.org/10.1186/s12887-025-05536-5>

**Prendergast M, Jones JS, and Hartman D**. 1994. Racemic epinephrine in the treatment of laryngotracheitis: can we identify children for outpatient therapy? *Am J Emerg Med* **12**:613-616. <http://dx.doi.org/10.1016/0735-6757(94)90024-8>

**Preutthipan A, Poomthavorn P, Sumanapisan A, Chinrat B, Thasuntia S, Plitponkarnpim A, and Chantarojanasiri T**. 2005. A prospective, randomized double-blind study in children comparing two doses of nebulized L-epinephrine in postintubation croup. *J Med Assoc Thai* **88**:508-512.

**Ritu, and Jhamb U**. 2020. Dexamethasone in prevention of postextubation stridor in ventilated children: A randomized, double-blinded, placebo-controlled trial. *Indian Journal of Critical Care Medicine* **24**:1230-1235. <http://dx.doi.org/10.5005/jp-journals-10071-23679>

**Rizos JD, DiGravio BE, Sehl MJ, Tallon JM, and Marx JA**. 1998. The disposition of children with croup treated with racemic epinephrine and dexamethasone in the emergency department. *Journal of Emergency Medicine* **16**:535-539. <http://dx.doi.org/10.1016/S0736-4679(98)00055-9>

**Rudinsky SL, Sharieff GQ, Law W, and Kanegaye JT**. 2015. Inpatient Treatment after Multi-Dose Racemic Epinephrine for Croup in the Emergency Department. *J Emerg Med* **49**:408-414. <http://dx.doi.org/10.1016/j.jemermed.2015.05.031>

**Scolnik D, Coates AL, Stephens D, Da Silva Z, Lavine E, and Schuh S**. 2006. Controlled delivery of high vs low humidity vs mist therapy for croup in emergency departments: a randomized controlled trial. *Jama* **295**:1274-1280. <http://dx.doi.org/10.1001/jama.295.11.1274>

**Singer OP, and Wilson WJ**. 1976. Laryngotracheobronchitis: 2 years' experience with racemic epinephrine. *Can Med Assoc J* **115**:132-134.

**Smith N, Giordano K, Thompson A, and DePiero A**. 2018. Failure of Outpatient Management With Different Observation Times After Racemic Epinephrine for Croup. *Clin Pediatr (Phila)* **57**:706-710. <http://dx.doi.org/10.1177/0009922817737075>

**Song HY, Kwon JH, Park SH, Kim MJ, Byun YH, and Paek SH**. 2024. Comparison of Croup Management Patterns between Pediatricians and Emergency Medicine Physicians: A Single Pediatric Emergency Department Study. *Journal of Clinical Medicine* **13**. <http://dx.doi.org/10.3390/jcm13206095>

**Sumboonnanonda A, Suwanjutha S, and Sirinavin S**. 1997. Randomized controlled trial of dexamethasone in infectious croup. *Journal of the Medical Association of Thailand* **80**:262-265.

**Super DM, Cartelli NA, Brooks LJ, Lembo RM, and Kumar ML**. 1989. A prospective randomized double-blind study to evaluate the effect of dexamethasone in acute laryngotracheitis. *Journal of pediatrics* **115**:323-329.

**Tan YT, Hartford EA, Barry D, Rutman LE, and Migita RT**. 2025. Standardization of Clinician Outcomes for Croup Using a Clinical Pathway. *Pediatr Emerg Care* **41**:554-559. <http://dx.doi.org/10.1097/pec.0000000000003385>

**Walsh PS, and Lipshaw MJ**. 2022. Diurnal Variation in Frequency and Severity of Croup in the Emergency Department. *Hospital Pediatrics* **12**:907-912. <http://dx.doi.org/10.1542/hpeds.2022-006682>

**Walsh PS, Zhang Y, and Lipshaw MJ**. 2023. Variation in Emergency Department Use of Racemic Epinephrine and Associated Outcomes for Croup. *Hosp Pediatr* **13**:167-173. <http://dx.doi.org/10.1542/hpeds.2022-006905>

**Winter M, Newth C, Castro C, Hotz J, and Ross P**. 2021. Continuous vaporized epinephrine safely administered to 138 children at a single institution. *Critical care medicine* **49**:494. <http://dx.doi.org/10.1097/01.ccm.0000729860.09480.06>

**Wyly DR, Berg K, Melanson A, Gripka M, Skoglund D, Blanco MV, and Nedved A**. 2025. Using a Clinical Pathway to Safely Reduce Transfers and Admissions for Croup in the Urgent Care. *Pediatr Emerg Care* **41**:635-640. <http://dx.doi.org/10.1097/pec.0000000000003396>
