# Supplementary material for: Nebulized epinephrine for croup in children: an updated systematic review
Source: Front Pediatr. 2026 Aug 26;14:1886410. doi: 10.3389/fped.2026.1886410 (PMC13553941; doi:10.3389/fped.2026.1886410)
Supplement: Supplementary file 3 [file Table3.docx]

**Supplementary Material 6 Studies included in the meta-analysis**

| **Author** | **Year** | | **Treatment Group (n)** | **Control Group (n)** | **Treatment Group Intervention** | **Treatment Dose** | **Control Group Intervention** | | **Control Dose** |
| --- | --- | --- | --- | --- | --- | --- | --- | --- | --- |
|  |  |  | |  |  |  |  |  | |
| Huazhen Wang | 2022 | 50 | | 50 | RE and BUD | RE 1ml, BUD 2ml Neb | BUD | BUD 2ml Neb | |
| Eghbali, A | 2016 | 87 | | 87 | LE and DXM | LE 0.5mg/kg Neb, DXM 0.6mg/kg IM (max 8mg) | DXM and Placebo | DXM 0.6mg/kg IM (max 8mg) | |
| Kristjánsson, S | 1994 | 25 | | 29 | RE | RE 0.5mg/kg Neb | Saline (placebo) | Saline 0.5mg/kg Neb | |
| Kuusela, A. L | 1988 | 19 | | 16 | RE and DXM | RE 0.05ml/kg Neb, DXM 0.6mg/kg IM | DXM and Placebo | DXM 0.6mg/kg IM | |
|  |  | 16 | | 21 | RE and Placebo | RE 0.05ml/kg Neb | Placebo |  | |
| Westley, C. R | 1978 | 10 | | 10 | RE | RE 0.5ml, preservative-free sterile water 3.5ml Neb | Saline | Saline 0.5ml, preservative-free sterile water 3.5ml Neb | |
| Taussig, L. M | 1975 | 7 | | 6 | RE | RE 0.5mg/kg Neb | Supportive care | Supportive care, including mist tent therapy, oxygen, adequate hydration, and antibiotics as needed. | |
| Gardner, H. G | 1973 | 10 | | 10 | RE | RE 0.5ml, saline: 3.5ml Neb | Placebo | Saline 4ml Neb | |

Racemic epinephrine: RE; L-epinephrine: LE; Dexamethasone: DXM; Budesonide: BUD; Nebulization: Neb; Intramuscular: IM
